# High-Resolution 3D Reconstruction of Human Oocytes Using Focused Ion Beam Scanning Electron Microscopy

**DOI:** 10.3389/fcell.2021.755740

**Published:** 2021-11-02

**Authors:** Zuzana Trebichalská, Jakub Javůrek, Martina Tatíčková, Drahomíra Kyjovská, Soňa Kloudová, Pavel Otevřel, Aleš Hampl, Zuzana Holubcová

**Affiliations:** ^1^Department of Histology and Embryology, Faculty of Medicine, Masaryk University, Brno, Czechia; ^2^TESCAN ORSAY HOLDING, a.s., Brno, Czechia; ^3^Reprofit International, Brno, Czechia

**Keywords:** electron microscopy, FIB-SEM, volume microscopy, 3D ultrastructure, human oocyte, oocyte maturation

## Abstract

The egg plays a pivotal role in the reproduction of our species. Nevertheless, its fundamental biology remains elusive. Transmission electron microscopy is traditionally used to inspect the ultrastructure of female gametes. However, two-dimensional micrographs contain only fragmentary information about the spatial organization of the complex oocyte cytoplasm. Here, we employed the Focused Ion Beam Scanning Electron Microscopy (FIB-SEM) to explore human oocyte intracellular morphology in three dimensions (3D). Volume reconstruction of generated image stacks provided an unprecedented view of ooplasmic architecture. Organelle distribution patterns observed in nine donor oocytes, representing three maturational stages, documented structural changes underlying the process by which the egg acquires developmental competence. 3D image segmentation was performed to extract information about distinct organelle populations, and the following quantitative analysis revealed that the mitochondrion occupies ∼ 4.26% of the maturing oocyte cytoplasm. In summary, this proof-of-concept study demonstrates the potential of large volume electron microscopy to study rare samples of delicate female gametes and paves the way for applying the FIB-SEM technique in human oocyte research.

## Introduction

With a typical spherical shape and a diameter of ∼110 μm, the egg constitutes the largest cell in the human body. When fertilized, its exceptionally voluminous ooplasm determines the cellular mass of a newly-formed zygote. Along with chromosome segregation, structural and biochemical changes in the oocyte’s cytoplasmic compartment are necessary to confer the oocyte with fertilization and developmental capacity (Watson, 2007). Although the female gamete’s influence over an embryo’s fate is generally acknowledged (Mtango et al., 2008), we still lack a clear understanding of human oocyte morphophysiology and cellular mechanisms underlying the development of a fertilizable egg.

Elucidating details about the spatial organization of complex ooplasm is critical to improving our knowledge of structural bases of cytoplasmic maturation. In our previous study (Trebichalská et al., 2021), we used transmission electron microscopy (TEM) to investigate the intracellular morphology of human oocytes maturing *in vitro*. Although TEM micrographs provided superior resolution, they pictured only two-dimensional (2D) projections of a small area within the researched sample. This limitation posed a challenge for ultrastructural data interpretation and morphometric measurements. The information about the 3D organization of cells and tissues can be obtained by digital reconstruction of orderly stacked 2D image series. However, individually trimmed ultrathin sections must be manually handled, carefully registered, and precisely aligned. This preparation principle makes serial-section TEM extremely arduous, time-consuming, and error-prone even in the hands of the experienced microscopist.

**FIGURE 1:**
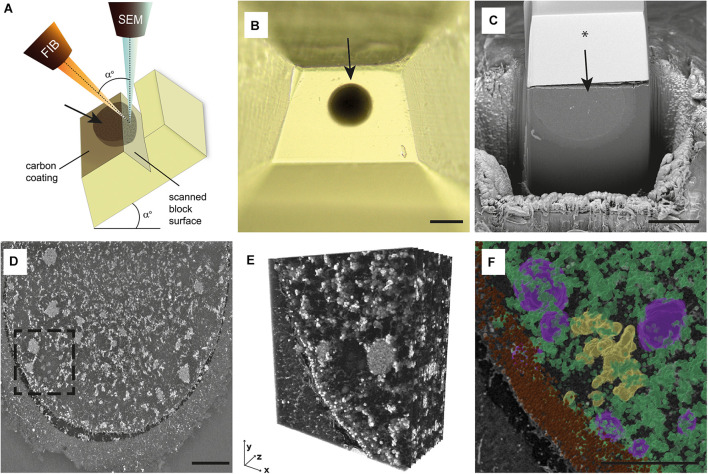
Overview of FIB-SEM imaging procedure and data processing. **(A)** The scheme of FIB-SEM setup. Both focused ion beam (FIB) and SEM (scanning electron microscope) columns are positioned in the microscope chamber, aiming at the scanned block surface’s coincidence point. The stage tilt corresponds to angle (a) between FIB and SEM column. The top of the surface of the resin block is coated with a carbon layer. Oocyte position is indicated with an arrow. **(B)** The top view of trimmed resin block with the sample embedded (arrow). **(C)** The trenched resin block with half of the oocyte (arrow). The block’s surface was coated with a thin carbon layer, and a silicon mask was placed over the sample (*). **(D)** The single grayscale image of MII oocyte surface scanned using FIB-SEM. **(E)** The 3D reconstruction of the spindle area [dashed rectangle in panel **(D)**]. **(F)** The 3D segmented and pseudocolored chromosomes (yellow), mitochondria (green), tubular endoplasmic reticulum (magenta), and cortical granules (brown) in the spindle area [dashed rectangle in **(D)**]. Scale bar: 100 μm **(B)**, 50 μm **(C)**, 10 μm **(D,F)**.

Focused Ion Beam Scanning Electron Microscopy (FIB-SEM) represents an alternative to notoriously laborious TEM tomography. It combines FIB-assisted ablation of a thin layer from the specimen’s block-face and SEM imaging of the freshly exposed surface (Figure 1A). By repeating milling and scanning cycles, the successive layers are of the sample are progressively eroded, and desired 3D volume recorded. This way, the dual-beam system automatically generates large sets of images with high resolution and an extensive field of view. Unlike TEM, which harvests the power of a high-energy electron beam passed through an ultrathin cross-section of the specimen, the SEM detects back-scattered and secondary electrons emitted from a limited depth beneath the ion-milled surface of the sample (Xu et al., 2017). Traditional FIB employs easily controlled gallium (Ga) ions for high precision material removal. Given the relatively low ion currents (≤100 nA), Ga FIBs are typically used for small samples not exceeding a few microns. Recently, FIB-SEM with xenon (Xe) plasma ion source gained popularity for its ability to achieve high ion currents (≤ 3 μA) capable of large-scale milling tasks. The higher ablation rate offered by Xe FIB makes imaging of large volumes more time-efficient (Hrnčíř et al., 2012).

Although initially developed for material sciences, FIB-SEM was applied to various biological specimens (Kizilyaprak et al., 2019). However, no study tested this technique’s applicability to image the inner structure of large and spherical mammalian oocytes. Here, we report the use of FIB-SEM tomography to visualize the 3D morphology of female gametes. Obtaining 3D images of human oocytes maturing *in vitro* allowed us to explore spatial relationships between intracellular components, track structural changes associated with cytoplasmic maturation and precisely quantify organelle abundance.

## Materials and Methods

### Human Oocytes

The analysis was performed on nine spare unfertilized oocytes derived from nine young and healthy egg donors (aged 22–29 years, average age 25.56 years) who participated in a clinical egg donation program between February 2018 and November 2019. The donors’ mature eggs were utilized for fertility treatment, and surplus immature oocytes were used for research purposes, provided that written informed consent was obtained. The research study was undertaken under ethical approval issued by the Ethics Committees of collaborating institutions.

Ovarian stimulation and oocyte collection were carried out as previously described (Trebichalská et al., 2021). Immature oocytes were incubated *in vitro* until they reached the defined developmental stage. Each oocyte’s meiotic status was determined based on the presence/absence of a germinal vesicle (GV), the first polar body (PB), and the meiotic spindle visualized by polarized light microscopy (PLM) (Holubcová et al., 2019). Oocytes featuring an intact prophase nucleus (GV oocytes) were fixed no later than 3 h after retrieval. Cells that showed a prominent PLM-detectable spindle signal but no PB 3–6 h of *in vitro* incubation were assigned to the MI oocyte category. Presence of a PB together with an MII spindle signal on the day after retrieval was considered a hallmark of MII oocyte maturity. Only normally appearing oocytes with no signs of dysmorphism were included in the study.

### Electron Microscopy

The oocytes were fixed overnight in 3% glutaraldehyde (AGR1012, Agar Scientific, Stansted, United Kingdom) in 0.1 M sodium cacodylate buffer (C0250, Sigma Aldrich, St. Louis, MO, United States) (pH 7.2–7.8) supplemented with 1% tannic acid (W304204, Sigma Aldrich, St. Louis, MO, United States) at room temperature. Following post-fixation with 1% osmium tetroxide (O5500, Sigma Aldrich, St. Louis, MO, United States) and 1.5% potassium ferrocyanide (P3289, Sigma Aldrich, St. Louis, MO, United States) for 1 h, the cells were individually embedded in 3% agarose (Sigma Aldrich, St. Louis, MO, United States), dehydrated and embedded in epoxy resin (44611-4, Durcupan, Sigma Aldrich, St. Louis, MO, United States). Each block of hardened resin was manually trimmed into a pyramid shape with an oocyte positioned at its top (Figure 1B). After being coated with a 15 nm thin carbon layer which reduced excessive charging, individual samples were inserted into a FIB-SEM equipped with an integrated Xe/Ga ion source (Tescan Amber (X)/Solaris X; Tescan Brno s.r.o., Brno, Czechia). The microscope stage was tilted to 55 degrees, corresponding to the angle between FIB and SEM columns (Figure 1A). Before starting image acquisition, the area around the sample was trenched, and the exposed block surface was smoothed out by FIB (Figure 1C). In order to reduce curtaining artifacts during FIB milling, a platinum protective layer was deposited on the surface of samples analyzed with Ga FIB, whereas silicon masks protected samples analyzed by Xe FIB (TESCAN TRUE-X sectioning method, described in detail in Hrnčíř et al. (2016). The FIB was operated at the energy of 30 keV and a beam current of 300 nA (Xe source) or 50–85 nA (Ga source) for trench etching and 100 nA (Xe source) or 20 nA (Ga source) for slicing and surface polishing. The SEM scanning was performed with accelerating voltage 3–5 kV, electron beam current 300–600 pA, working distance 5–6 mm, dwell time 10–32 us/px, and pixel size 30–40 nm (xy)/40–100 nm (z).

### Image Analysis

The generated image stacks were filtered using a 3D median in Fiji software (Schindelin et al., 2012). The image stacks were imported into Amira software version 2019.3 (Thermo Fisher Scientific, Waltham, Massachusetts, United States) for post-processing, The pixels that represented the mitochondria were automatically detected using the adjusted threshold option (the threshold was applied manually based on visualization of gray-level histogram). The individual z-sections were proofread to ensure the accuracy of software-operated detection, and the false-positive labels were manually removed. The other microstructures (chromosomes, ER and cortical granules) were segmented manually based on their appearance and pseudocolored. To generate animations, the segmented binary masks were converted into 3D models using Generate surface module. The scanned oocyte volume and total mitochondrion volume were quantified by Amira software using the segmented volume data. The percentage of mitochondria was calculated per imaged oocyte volume.

## Results

To examine the suitability of the FIB-SEM technique for volumetric imaging of large female gametes, we used a total of nine human oocytes fixed at three consecutive stages of egg maturation: three germinal vesicle (GV), three metaphase I (MI), and three metaphase (MII) oocytes. The samples were processed using routine TEM protocol (Trebichalská et al., 2021), but instead of manual ultramicrotome sectioning, whole oocyte-containing resin blocks were individually placed into the FIB-SEM microscope chamber and subjected to automated serial imaging (Figure 1A). To minimize the risk of excessive charging, the sample surface was pre-conditioned with carbon coating. We tested both Xe and Ga FIB-SEM systems and compared their performances to determine optimal imaging regimens (technical parameters of each experiment are detailed in Table 1). The advantage of Xe FIB was that a high current beam reduced the time required for digging a primary trench around the area of interest. However, ion currents > 300 nA put delicate samples at risk of considerable heat stress. According to our experience, currents up to 100 nA are sufficient to slice through even large volumes in a reasonable time. An automated Ga ion source reheating allowed us to run tomography without interruption, and additional functions such as automated drift correction and autofocus made the acquisition process robust and reliable. Our efforts led us to conclude that, following appropriate optimization, both Xe- and Ga-based FIB-SEM systems are suitable for large volume microscopy of resin-embedded female gametes.

**TABLE 1:** The imaging conditions of individual human oocyte samples.

				FIB settings			SEM settings				Dataset characteristics					
Maturation stage	Sample ID	FIB-SEM microscope	Imaging time (h)	FIB type	Trench milling (nA)	Slicing (nA)	Detector type	Voltage (kV)	Beam current (pA)	Dwell time (μs/px)	Number of slices	Pixel XY (nm)	Pixel Z (nm)	XY pixel size	XY area (nm)	Z area (nm)
GV	MU-59	Amber X	39,8	Xe	300	100	LE-BSE	5	300	32	322	30	90	3,165 × 1,452	94,950 × 43,650	28,980
	MU-107	Solaris X	43,5	Xe	300	100	LE-BSE	4	300	32	222	40	100	4,224 × 1,674	168,960 × 66,960	22,200
	MU-158	Amber	49,4	Ga	50-85	20	LE-BSE	5	500	10	636	30	90	4,899 × 2,394	146,970 × 71,820	57,240
MI	MU-73	Amber	89,8	Ga	50-85	20	LE-BSE	5	600	10	1,294	30	90	4,124 × 2,228	123,720 × 66,840	116,460
	MU-75	Amber	76,2	Ga	50-85	20	LE-BSE	5	300	10	1,125	30	90	4,416 × 2,622	132,480 × 78,660	101,250
	MU-77	Solaris X	5,3	Xe	300	100	LE-BSE	4	300	32	120	40	100	1,364 × 1,462	54,560 × 58,480	12,000
MII	MU-89	Amber X	75,3	Xe	300	100	LE-BSE	3	400	30	237	30	90	5,043 × 3,315	151,290 × 99,450	21,330
	MU-108	Amber X	22,7	Xe	300	100	Axial	5	300	32	451	40	100	2,461 × 1,715	98,440 × 68,600	45,100
	MU-113	Amber X	55,1	Xe	300	100	Axial	3	300	19,2	494	40	40	3,100 × 2,152	124,000 × 86,080	19,760

The image acquisition time using FIB-SEM depends on the microscope properties and operating conditions, namely scanning speed, resolution, and the covered volume. In our hands, the continuous processing of bulk specimens lasted 5–90 h, and the total volume we imaged ranged from 3.83 × 10^4^ to 1.06 × 10^6^ μm^3^ (Table 1). In some experiments, we cut out the sample’s top part to speed up trenching and expose the cell’s central segment we aimed to record (Figures 1B,C). Gradual improvement of imaging conditions allowed us to visualize up to ∼65% of the human oocyte volume with 40–100 nm z-resolution. Although the individual grayscale scans of sample surface did not show the same level of detail as TEM micrographs (Supplementary Figure 1), the digital reconstruction of the FIB-SEM acquired image stacks (120–1,294 slices) gave us the possibility to watch through the large volume of ooplasm and study mutual interactions of subcellular structures in both vertical and horizontal axis (Figures 1D–F and Supplementary Videos 1, 2).

**FIGURE 2:**
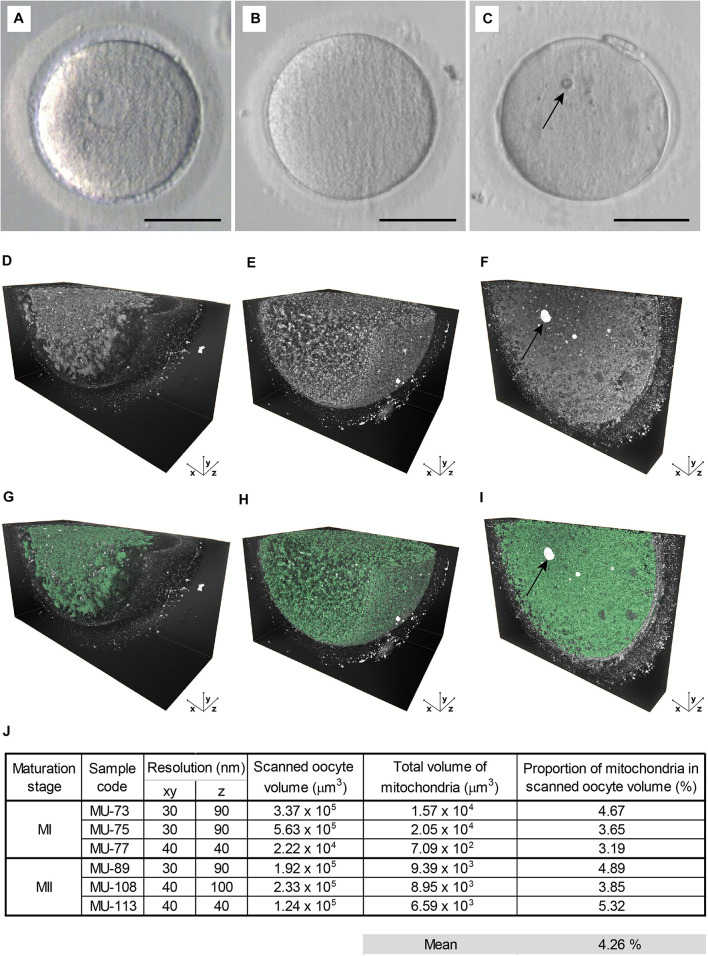
Large volume electron microscopy of maturing oocytes. **(A–C)** The live appearance of representative **(A)** germinal vesicle (GV), **(B)** metaphase I (MI), and **(C)** metaphase II (MII) oocyte in transmitted light. Scale bar: 50 μm. **(D–F)** The histogram-enhanced 3D image of **(D)** GV, **(E)** MI, and **(F)** MII oocyte. **(G–I)** The 3D image in **(G)** GV, **(H)** MI, and **(I)** MII oocyte with mitochondria segmented (green). An arrow indicates prominent cytoplasmic inclusion **(C,F,I)**. **(J)** Quantification of a total mitochondrial volume in analyzed MI and MII oocyte samples.

The most prominent ultrastructural features found in oocyte cytoplasm were spherical or ovoid-shaped mitochondria (∼450 nm in diameter), aggregates of the endoplasmic reticulum (ER) (∼5 μm in diameter), and cortical granules (∼300 nm in diameter) docked beneath the oolema (Figures 1D,E). In MI and MII oocytes, chromosomes and microtubule bundles could be recognized in the meiotic spindle area tightly enclosed by organelle-rich cytoplasm (Figures 1D–F and Supplementary Videos 1, 2). These findings are in line with previous conventional TEM studies of human oocyte ultrastructure (Sundström et al., 1985; Sathananthan, 1994; Bianchi et al., 2015; Trebichalská et al., 2021). Due to insufficient contrast, we could not discern the edges of ER sacs which form characteristic “necklace” complexes with mitochondria (Supplementary Figure 1), but the clusters of tubular ER were found to be scattered in the cytoplasm of the majority (5/6) MI and MII oocytes analyzed (Supplementary Figure 1 and Supplementary Videos 1, 2). Whereas in TEM micrographs, only a few mitochondria could be detected in the vicinity of ER, FIB-SEM revealed that enlarged mitochondrial clumps surrounded ER aggregates. Unlike much bigger cytoplasmic plaques (sER disks) (Sá et al., 2011), these relatively small ER clusters are not perceptible during routine stereomicroscope examination. Notably, all but one sample exhibited prominent electron-dense specs with a diameter reaching up to 7 μm (Figures 2C,F,I and Supplementary Video 1). Such cytoplasmic inclusions, referred to as refractile bodies due to their autofluorescence (Otsuki et al., 2007), are common in clinically utilized human eggs. As documented in our previous TEM study, these microstructures appear to deposit degraded intracellular material accumulated in long-lived female gametes (Trebichalská et al., 2021). Our 3D image data added support to existing evidence on morphological peculiarities of human oocytes. Furthermore, volumetric reconstruction of FIB-SEM-generated image stacks provided better insight into topological properties of subcellular structures and their spatial relationships than flat 2D micrographs.

Next, we compared 3D ultrastructural patterns of GV, MI, and MII oocytes (Figures 2A–C) to assess how the maturation stage affects compartmentalization of the ooplasm. Volumetric data showed that the cortical area of prophase-arrested GV oocytes was deprived of membraneous organelles (Figure 2D and Supplementary Video 2). On the contrary, in the close-to-anaphase MI oocytes and MII-arrested eggs, the detectable intracellular structures were evenly distributed throughout the cell mass (Figures 2E,F and Supplementary Video 2), indicating that the meiotic resumption triggered organelle relocation toward the cell’s periphery. Hence, the FIB-SEM image analysis confirmed our previous assumption that oocyte maturation involves territorial rearrangement of cytoplasmic components with massive organelle redistribution occurring during the MI stage, before PB extrusion (Trebichalská et al., 2021).

To further evaluate spatial relationships of individual organelles, we subjected generated FIB-SEM datasets to software-enhanced 3D image segmentation (Figure 1F and Supplementary Video 2). The detected microstructures were volume-rendered and pseudocolored. For quantitative measurements, we focused on the most abundant organelles, the mitochondria, that exhibited typical morphology and sufficient contrast in all datasets (Figures 2G–I). The mitochondria volume was counted in MI and MII oocytes characteristic with uniform distribution of organelles, whereas GV oocytes were excluded to avoid bias caused by unequal representation of the organelle-rich perinuclear area in the subvolumes of ooplasm we visualized. The quantitative analysis of the 3D image data indicated that, in our samples of maturing human oocytes, mitochondrion occupies approximately 4.26% cytoplasm volume (Figure 2J). This result derived from densely recorded 3D datasets provide a more accurate estimate than the previous TEM stereological studies using 2D image data (Pires-Luís et al., 2016; Coelho et al., 2020).

## Discussion

To the best of our knowledge, this is the first study investigating the ultrastructure of human oocytes in 3D. We tested different microscopes systems and found both Ga- and Xe-based FIB-SEM suitable for large volume microscopy of female gametes. Using the automated slicing-scanning approach in we were able to (1) reconstruct substantial volumes of ooplasm, (2) examine spatial relations and distribution of microstructures, and (3) quantify organelle population in rare samples of maturing human oocytes. Volumetric reconstruction of FIB-SEM acquired image stacks allowed us to appreciate the 3D aspect of subcellular components’ organization and complexity of ooplasmic architecture. By analysis of maturing oocytes’ 3D ultrastructure, we enhanced our understanding of cytoplasmic maturation. It can be assumed that leveraging the power of 3D imaging would upgrade studies assessing how alteration of culture conditions, experimental perturbation, and vitrification affect human oocyte morphology. Moreover, the detailed knowledge of the female gametes’ inner morphology has vital implications for developing experimental and therapeutical strategies involving oocyte microsurgery, such as spindle or cytoplasm transfer for mitochondrial replacement therapy (Darbandi et al., 2017).

Since the procurement of human female gametes for research is limited to small numbers, maximizing the amount of information obtained from a single cell is crucial. Our work illustrates that volumetric imaging combined with 3D image analysis increases the yield of morphological data from precious biological samples, such as human oocytes. To examine the ultrastructural basis of cytoplasmic maturation, we used surplus *in vitro* maturing oocytes derived from young and healthy egg donors. Although the impact of ovarian stimulation, late response to hormonally priming, and *in vitro* maturation on egg quality cannot be excluded, these samples are more representative of normal egg physiology than reproductively aged and failed-to-be-fertilized female gametes derived from patients struggling with infertility.

The volumetric image data presented in this paper complemented our findings from the previous TEM study (Trebichalská et al., 2021). The fact that we found no major discrepancy between TEM and FIB-SEM image data supports the notion that this sample type is suitable for systematic research of inherently variable female gametes. Consistent fixation and sample processing protocol enable straightforward comparison of image data generated by the two approaches. Our results showed that TEM provides better image contrast and thus remains a gold standard for scrutinizing fine morphological details. Yet, the enormous workload required for manual trimming and individual imaging of ultrathin sections is impractical for large volume ultrastructural analysis. On the contrary, the precisely sectioned and automatically scanned FIB-SEM images with a large field of view are well suited for mapping global ooplasmic patterning. The 3D reconstruction of a large number of FIB-SEM images shows a more complete picture of the cell interior than a collection of individually trimmed cross-sections. Conveniently, FIB-SEM instruments offer flexibility to switch from large-volume tomography to high-resolution 3D imaging of the targeted region of interest in the course of imaging experiment. Compared to confocal microscopy, commonly used to locate oocyte’s intracellular structures, FIB-SEM offers much higher z-resolution enabling accurate quantification of submicrometer-sized organelles. Unlike fluorescent imaging, which relies on specific probe availability and a limited number of channels, FIB-SEM allows simultaneous visualization of multiple contrasted microstructures without the risk of signal bleaching introduced during intense 3D imaging.

Besides a nanometer-scale axial resolution and wide range of magnifications, *in situ* manipulation and full automation represent the FIB-SEM technique’s main advantages. The common artifacts introduced during manual ultramicrotoming, such as folds, scratches, and contaminations, are avoided. However, considering this method’s irreversibly destructive nature, imaging protocol optimization is required to minimize the risk of thermic distortion, excessive charging, and block-face curtaining, especially when working with rare samples. The technological advancements are aimed to balance the trade-off between resolution and speed of imaging. Future developments in precision instrumentation, big data management tools, and artificial intelligence-assisted software solutions promise to enhance FIB-SEM imaging’s time efficiency and facilitate 3D image data analysis.

In conclusion, we present the FIB-SEM technique as a novel research tool that extends the palette of techniques suitable for investigating human oogenesis. We believe that the prospect of visualizing whole oocyte volume in great detail would stimulate further studies looking into the inner workings of the cell our life starts with.

## Data Availability Statement

The raw data supporting the conclusions of this article will be made available by the authors, without undue reservation.

## Ethics Statement

The studies involving human participants were reviewed and approved by (1) Ethics Committee of Faculty of Medicine, Masaryk University, and (2) Ethics Committee of Retrofit International. The patients/participants provided their written informed consent to participate in this study.

## Author Contributions

ZT performed sample collection, fixation, and processing for electron microscopy, analyzed generated 3D data, designed figures, and wrote the first draft of the manuscript. JJ supervised the automated FIB-SEM imaging and optimized imaging setup. MT contributed to 3D image data analysis and generation of animations. DK performed a morphological assessment and polarized light microscopy of donated oocyte samples. SK was in charge of the administration of informed consents. PO supervised the recruitment of the egg donors and ovarian stimulation. AH provided discussions over the project’s design and proofread the manuscript. ZH designed the project and secured funding, established collaboration with clinical and industrial partners, analyzed the data, and wrote the final version of the manuscript. All authors read and approved the submitted version of the manuscript.

## Conflict of Interest

JJ is employed by TESCAN ORSAY HOLDING, the manufacturer of the FIB-SEM microscopes used in this study. However, the study did not receive any funding from the industrial partner. The remaining authors declare that the research was conducted in the absence of any commercial or financial relationships that could be construed as a potential conflict of interest.

## Publisher’s Note

All claims expressed in this article are solely those of the authors and do not necessarily represent those of their affiliated organizations, or those of the publisher, the editors and the reviewers. Any product that may be evaluated in this article, or claim that may be made by its manufacturer, is not guaranteed or endorsed by the publisher.

## Supplementary Material

Supplementary Figure 1 Comparison of TEM and FIB-SEM micrograph. **(A)** A cross-sectional TEM image of MII oocyte [reproduced from Trebichalská et al. (2021)] and **(B)** a single FIB-SEM of MI oocyte (3D reconstruction of an image stack is shown in Supplementary Video 1). Arrows indicate meiotic spindles, aggregates of tubular ER (+) are visible in the cytoplasm **(A,B)**. “Necklace-like” complexes of mitochondria and endoplasmic reticulum (ER) (^∗^) can be observed in TEM images **(A)** but are difficult to be discerned in FIB-SEM images **(B)**. Scale bar, 10 μm.

Supplementary Video 1 3D reconstruction of the FIB-SEM-acquired image stack. FIB-SEM-acquired stack of grayscale(-inverted) images and 3D reconstructions of MI/MII oocyte volume. Arrow indicates MI spindle with individual microtubules visible.

Supplementary Video 2 3D visualization of distinct organelle populations in maturing oocytes. Organelle segmentation in maturing oocytes. Representative examples of GV, MI, and MII oocytes: mitochondria (green), smooth ER (magenta), chromosomes (yellow), and cortical granules (brown).
