# Supplementary figures and images for: High-Resolution 3D Reconstruction of Human Oocytes Using Focused Ion Beam Scanning Electron Microscopy
Source: Front Cell Dev Biol. 2021 Nov 2;9:755740. doi: 10.3389/fcell.2021.755740 (PMC8593100; doi:10.3389/fcell.2021.755740)

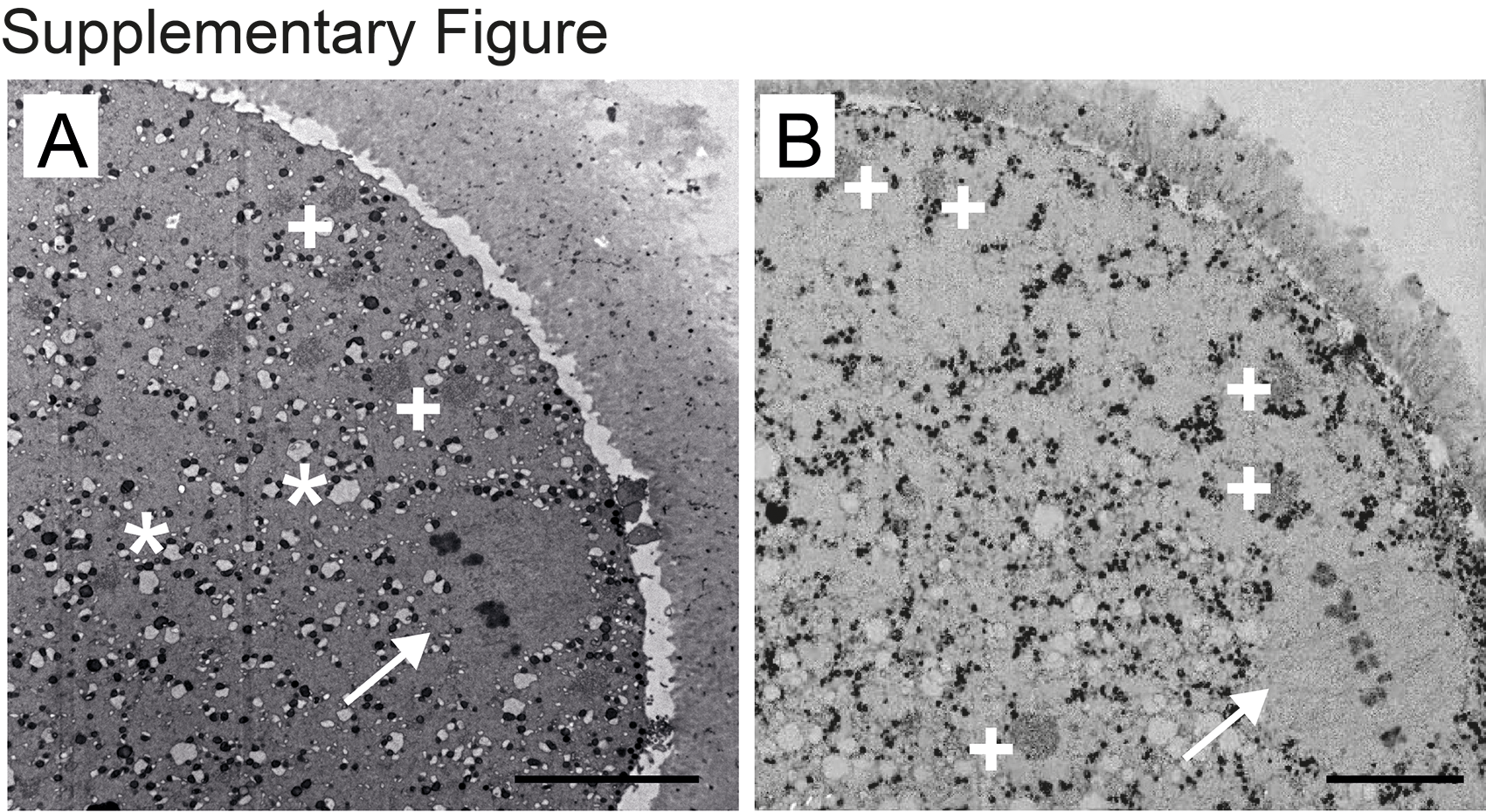

Supplement: Supplementary Figure 1 — Comparison of TEM and FIB-SEM micrograph. (A) A cross-sectional TEM image of MII oocyte [reproduced from Trebichalská et al. (2021)] and (B) a single FIB-SEM of MI oocyte (3D reconstruction of an image stack is shown in Supplementary Video 1). Arrows indicate meiotic spindles, aggregates of tubular ER (+) are visible in the cytoplasm (A,B). “Necklace-like” complexes of mitochondria and endoplasmic reticulum (ER) (∗) can be observed in TEM images (A) but are difficult to be discerned in FIB-SEM images (B). Scale bar, 10 μm. [file Image_1.TIF]
